# Barriers to Integration of Traditional and Complementary Medicine in Supportive Cancer Care of Arab Patients in Northern Israel

**DOI:** 10.1155/2012/401867

**Published:** 2011-12-01

**Authors:** Eran Ben-Arye, Mariana Steiner, Khaled Karkabi, Tamar Shalom, Levava Levy, Ariela Popper-Giveon, Elad Schiff

**Affiliations:** ^1^Integrative Oncology Program, The Oncology Service and Lin Medical Center, Clalit Health Services, Haifa and Western Galilee District, Haifa 35152, Israel; ^2^Complementary and Traditional Medicine Unit, Department of Family Medicine, Faculty of Medicine, Technion-Israel Institute of Technology, Haifa 32000, Israel; ^3^The Carmel Medical Center, Clalit Health Services, Haifa 34362, Israel; ^4^School of Public Health, Faculty of Social Welfare & Health Sciences, University of Haifa, Haifa 31905, Israel; ^5^David Yellin Academic College of Education, Jerusalem 91035, Israel; ^6^Department of Internal Medicine and Complementary and Integrative Surgery Service, Bnai Zion Medical Center, Haifa 33394, Israel

## Abstract

In 2008, an Integrative Oncology Program (IOP), aiming to improve patients' quality of life during chemotherapy and advanced cancer, was launched within the Clalit Health Organization's oncology service at the Lin Medical Center, Haifa, Israel. The IOP clinical activity is documented using a research-based registry protocol. In this study, we present an analysis of the registry protocol of 15 Arab patients with cancer who were referred to the IOP. Analysis of patients' reported outcomes using the Edmonton Symptom Assessment Scale suggests that integrative medicine care improves fatigue (*P* = 0.024), nausea (*P* = 0.043), depression (*P* = 0.012), anxiety (*P* = 0.044), appetite (*P* = 0.012), and general well-being (*P* = 0.031). Barriers to integration of traditional and complementary medicine in supportive care of Arab patients are discussed followed by six practical recommendations aimed at improving accessibility of patients to integrative supportive care, as well as compliance with treatments.

## 1. Introduction

The Middle East is represented by a rich spectrum of indigenous traditional schools of medicine modeled on a mosaic of social, religious, and spiritual perspectives. Testaments to the amalgam of indigenous roots of medical knowledge can be found in current ethno botanical surveys which document the use of herbs for cancer care in the regions of Israel [1], Syria [2], and the Palestinian Authority [3]. In a survey of Islamic and Jewish traditional medicine historical texts, scholars from Israel, Egypt, and Turkey identified 44 herbs associated with cancer care [4]. In a subsequent international study, a multidisciplinary team of researchers from Israel, the Palestinian Authority, Jordan, Egypt, Morocco and Turkey identified 143 articles on traditional/complementary medicine and cancer care that had been published on medline in 12 Middle Eastern countries [5].

 Several studies documented the significant use of complementary medicine (CM) by patients in the Middle East during chemotherapy (Israel, 49%) [6] and radiotherapy (Turkey, 44%) [7] and in subsets of patients with cancer: pediatric (Lebanon, 15%; Turkey, 77%; Israel, 61%) [8–10], gynecological (Turkey, 38%; Israel, 63%) [11, 12], and breast cancer (Israel, 44%) [12].

In Israel, the concept of CM integration within conventional care has been significantly studied among the Arab population in northern Israel. Ben-Arye and his colleagues [13] have studied the prevalence of CM use and attitudes toward its integration among 3840 patients in 7 primary care clinics operated by Clalit Health Service (CHS) and found that respondents in both groups significantly supported CM integration within primary care clinics.

Following this study, the Haifa and Western Galilee District of CHS initiated a study in 2007 to examine the possibility of CM integration within its oncology service (OS). In 2008, an Integrative Oncology Program (IOP) was launched as a free-of-charge clinical service aiming to improve patients' QOL during chemotherapy and advanced disease state. The IOP is based on a multidisciplinary team that includes physicians and practitioners that are dual trained in conventional care as well as CM. The IOP team provides a wide spectrum of traditional and CM modalities which include nutritional counseling (diet and supplements), herbal medicine, mind-body and touch therapies, acupuncture, anthroposophic medicine, homeopathy and spiritual care. In this paper, we present data regarding Arab patients receiving integrative treatment offered by the IOP during the years 2009–2011. We examine the needs and concerns of Arab patients who were referred to the IOP and explore difficulties and barriers to the provision of CM to this group of patients in integrative setting. Based on these observations, we advocate practical recommendations that may facilitate a cross-culturally sensitive approach that will resonate with Arab patients' expectations and needs in similar integrative health settings.

## 2. Materials and Methods

### 2.1. Registry Protocol Data Collection

The IOP clinical activities are documented in a research-based registry protocol (RP) approved by the IRB of the Carmel Medical Center, Haifa, Israel. The RP monitors patients' needs and concerns, symptom and QOL assessment, and prospective evaluation of clinical outcomes. In addition, the RP documents referral patterns, CM practitioner-patient-oncologist communication aspects, and assessment of the patient's, oncologist's, and the integrative physician's perspectives regarding the impact of the integrative intervention on the patient's well-being.

**Figure 1:**
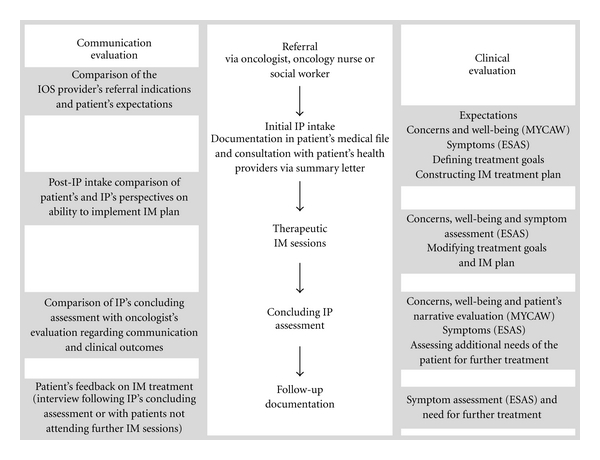
Flowchart of clinical and communicational evaluation along the sequence of integrative sessions within the Integrative Oncology Program (IOS: integrative oncology service; IP: integrative physician; IM: integrative medicine).

Figure 1 illustrates the flowchart beginning with the patient's referral to the IOP and concluding with follow-up assessments of the integrative process. Referral to the IOP may be initiated by the patient's oncologist, oncology nurse, or social worker and is limited to patients treated within the oncology service during chemotherapy and/or advanced cancer. Following the referral, an initial integrative medical intake interview is scheduled for one hour with an integrative physician (IP) who assesses the patient's expectations regarding CM, previous experience with traditional, alternative or CM, as well as the patient's narrative and outlook regarding diagnosis, treatment, coping, and well-being. The severity of symptoms, concerns, and expectations are evaluated by the IP using the Edmonton Symptom Assessment Scale (ESAS) and Measure Yourself Concerns and Wellbeing (MYCAW) questionnaires and a detailed bio-psychospiritual assessment. The session is typically concluded with outlining of the treatment goals that are shared by the patient and IP, followed by construction of a preliminary treatment plan tailored to the patient's outlook (concerns, symptoms, willingness to experience CM modalities, etc.) and level of evidence (efficacy, safety, possible interactions with chemotherapy, etc.). Each visit is recorded by the IP in the patient's medical file, and a clinical summary is distributed to the patient's healthcare providers (oncologist, nurse, family practitioner, social worker, etc.). Patients are typically scheduled for therapeutic integrative medicine (IM) sessions that may include a variety of CM modalities (e.g., nutritional and herbal counseling, acupuncture, mind-body, and manual therapies) provided from once every week to once every 2-3 weeks. Prior to therapeutic sessions, additional clinical assessment is conducted, aimed to modify, if necessary, the treatment goals and plan. Following 2–4 months of treatment, a concluding clinical assessment is performed with the use of ESAS and MYCAW questionnaires. More therapeutic sessions are provided, if deemed necessary, for patients with advanced cancer or for those receiving adjuvant chemotherapy. Such sessions are also regularly monitored. Figure 1 illustrates supplemental evaluation documented within the registry protocol regarding themes of patients' expectations and communication with healthcare providers. Follow-up evaluations include a self-administered questionnaire completed by the IP at the conclusion of the first medical intake, semistructured telephone interviews with patients conducted by a researcher following the first intake and after the concluding clinical evaluation and a questionnaire administered to the patient's oncologist following the concluding evaluation. In this evaluation process, the patient's and clinician's perspectives are independently compared regarding expectations, satisfaction with treatment and communication, as well as needs that were not fully addressed.

### 2.2. Assessment Methods

Assessment questionnaires include the following.

(a) MYCAW is an individualized questionnaire constructed and validated by Paterson et al. [14] for evaluating outcomes in cancer support care that includes complementary therapies [15]. Participants were asked to enumerate one or two concerns and, using a seven-point scale, to score these concerns and their general feeling of well being. The follow-up questionnaire also includes the following open question: “Reflecting on your time with this Centre, what were the most important aspects for you?”

(b) ESAS is a questionnaire developed for assessing the symptoms of patients receiving palliative care [16], as well as for assessing outcomes in an integrative oncology context [17]. It consists of an 11-point numerical rating scale for self-reporting of nine common symptoms of cancer, with a 10th scale for assessing the feeling of well-being. Both MYCAW and ESAS questionnaires were linguistically validated to Hebrew using bidirectional translation from their English origin to Hebrew and vice versa.

(c) Questionnaires administered to the IP and/or oncologist and/or patients were developed by the authors following a comprehensive literature review of patients' needs, concerns, and expectations regarding CM in the oncology setting and interviews with 24 patients in different phases of oncology treatment and with 61 health care providers (HCPs) and CM practitioners. Afterwards, a focus group, composed of 5 patients in different phases of cancer treatment, was used to refine the questionnaire and improve its comprehensibility. The focus group participants varied in age, sex, education, health status and CM use. Based on their feedback, the questionnaire was revised and sent for reappraisal to 7 of the HCPs. The final version of the 20-question questionnaire was administered by the IP following initial consultations. The questionnaire consists of 9 limited-choice questions (yes, no, other, or not relevant), 4 multiple-choice questions, one open question, and 6 questions that use a Likert-like scale. Independent researchers interviewed patients, following the IP's initial and concluding assessment, using a similar form of the questionnaire filled in by the IP. In addition, a researcher typically phoned to interview patients no longer attending the IM sessions. Another questionnaire was administered to the patient's oncologist following the IP's concluding assessment. This shortened questionnaire format consists of 4 questions including 3 that utilize a Likert-like scale.

Data was evaluated using the SPSS software program (version 18; SPSS Inc., Chicago, Ill). Wilcoxon Signed Ranks Test and paired *t*-test were used to detect differences before and after the treatment scores in MYCAW and ESAS questionnaires.

## 3. Results and Discussion

Participation in the registry protocol-based IOP research was limited to patients in the CHS Haifa and Western Galilee OS during chemotherapy or advanced active disease. The total number of new patients referred to the OS ranges from 800 to 1000 per year. Data regarding the cultural and religious characterization of this newly referred population was not available, nor was data related to the population of patients who theoretically meet the inclusion criteria for referral to the IOP. The best culture-related data available was obtained from analysis of nurse oncology intakes (NOI) which were performed prior to beginning chemotherapy in patients receiving intravenous adjuvant, neoadjuvant, or palliative chemotherapy for the first time in their life. The NOI-based data was collected and analyzed starting from 14/7/2009 (parallel to the launching of the registry protocol) and up to 14/7/2011.

 Based on the patient's self-report of spoken language during the NOI, we divided patients into two groups: Arabs (patients speaking Arabic solely or in addition to Hebrew or other languages) or non-Arabs (patients speaking Hebrew or other languages but not Arabic). In cases not determined by the language criterion, we assigned the patient to one of these groups according to the father's name. Five hundred thirty-one patients were thus grouped based on the NOI data during the two-year study, of whom 103 were Arabs (19.4%) and 428 non-Arabs (80.6%). This data may not reflect the entire population of patients who could have been referred potentially by the OS health providers to the IOP, for the following reasons: (a) patients with recurrent disease treated with chemotherapy initiated prior to July 2009 (about 42.4% of patients in the OS are receiving chemotherapy for recurrent disease); (b) patients receiving oral chemotherapy, biological or hormonal therapy; (c) patients receiving palliative care with no chemotherapy initiated after July 2007.

**Figure 2:**
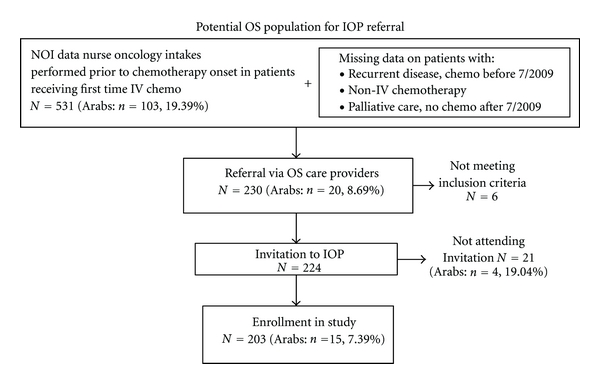
Algorithm of study recruitment (in brackets the proportion of Arab patients).

During this two-year period, 230 of the 531 patients (recorded in NOI data) were referred to the IOP by the OS oncologists, oncology nurses, or OS social workers. Of these referrals, 224 met the inclusion criteria for IOP admission and enrollment in the registry protocol-based research. Of the 224 patients invited for IOP assessment, 203 (response rate 90.6%) participated in the IP's initial intake and provided written consent to participate in the research protocol study. Of the 203 study subjects, who were monitored in the registry protocol, 15 were Arabs (7.39%) and 188 were non-Arabs (92.61%).  Figure 2 illustrates an algorithm of the study's recruitment and illustrates the proportion of Arab versus non-Arab patients along the funnel leading to the registry protocol tracking.

Of the 21 patients who were referred to the IOP but did not attend the IP's intake, 4 were Arabs and 17 non-Arabs. The main reason for nonattendance was difficulty in scheduling appointments during progressive treatment.

### 3.1. Registry Protocol Demographics of Arab Patients

The demographic characteristics of Arab patients enrolled in the integrative registry protocol within the OS are presented in Table 1. This population is characterized by female predominance (11/15, 73.3%), mean age of 52.4 years (range 22–77), and variety of religions (6 Muslim, 5 Christian, and 4 Druze). Most patients reside in cities (9 urban, 4 rural, and 2 semiurban) which are often very far from the OS located in Haifa (4 patients live in zone 1 (Haifa), 1 patient in zone 2 (up to 20 km from Haifa), and 10 in zone 3 (>20 km from Haifa)). Of the 15 patients, 9 were diagnosed with localized disease and were receiving adjuvant chemotherapy following surgery and 6 had advanced disease and were receiving palliative chemotherapy. Patients' cancer sites included the following: breast (6), colon (3), stomach (1), lung (1), bladder (1), testicles (1), mesothelioma (1), and an undiagnosed site (1).

### 3.2. Assessment of Referral, Expectations, and Communication during and following IP Intake

Referral to IOP was performed via health care practitioners' (HCPs) structured referral letters that specify the indications for referral to the integrative treatment. Of the 15 referrals, 9 were administered by oncology nurses, 4 by oncologists, and 3 by the OS social workers (one referral was administered by both nurse and social worker).

Nine of the 15 patients (60%) reported previous use of traditional/CM in the context of cancer care. Although the IP specifically inquired about cancer-related CM use at the beginning of the medical intake, the majority of patients disclosed CM use only when asked for the second or third time towards the end of the interview. In these cases, disclosure was often related to the patient becoming aware that the definition of cancer-related CM also includes QOL improvement and symptom management rather than curing cancer.

General expectation to reduce chemotherapy side effects was the leading referral indication (6 referrals), followed by more specific expectations concerning symptoms such as anxiety (5), fatigue (3), and vomiting (2). Compared to indications for referrals, patients expressed additional expectations from the IP: what to eat and which herbs to use (4), how to alleviate fatigue and strengthen their condition (4), and how to improve management of pain (3), emotional state (2), and other symptoms. Expectation analysis of each of the 15 referrer-patient couples revealed that matching of expectations was largely evident in only four pairs for specific symptoms that need to be addressed by the IP (e.g., improving urination, bitter taste, stomatitis, and other gastrointestinal symptoms). Compared to HCPs, patients expressed more concrete expectations from the IP (e.g., specific symptom improvement versus general reduction of side effects) and in some cases anticipated outcomes beyond QOL improvement such as cancer cure and recurrence prevention.

Assessment of HCP-IP communication following the IP's initial intake revealed that the IP addressed a medical letter to all 15 patients' oncologists and to 14 nurses, 14 social workers, and 9 family physicians. Typically, these letters were referred to 3-4 of the patient's HCPs and often responded to by at least two HCPs.

Patient's and IP's evaluation were based on questionnaires completed by the IP following the initial visits and semistructured interviews with patients that were conducted by an independent researcher. Of the 15 patients interviewed, 5 anticipated difficulty in implementing the therapeutic plan as presented by the IP in the first visit. In contrast, the IP expected difficulty in 12 of the patients, mainly due to limited accessibility to the clinic and an impression that these patients doubted the benefits of the treatment. In 12 out of 15 patients there was incongruence between the patient and IP regarding perceived difficulty in implementing the treatment plan. This patient-IP mismatch is also evident in 9 patients regarding satisfaction following the initial visit. Compared to patient evaluations, IP scores were higher in 7 of these 9 pairs.

### 3.3. Clinical Assessment in Initial IP Intakes

Evaluation of the severity of patients' two main concerns was performed both in the initial IP intake and the concluding session that followed several therapeutic sessions. This evaluation was based on the MYCAW questionnaire which followed symptom assessment based on the ESAS questionnaire and an open interview with the IP. Leading concerns consisted of gastrointestinal symptoms (9) including nausea/vomiting, digestion, bitter taste, and other mouth symptoms; emotional distress (5); fatigue (5); pain/neuropathy (3); other symptoms. The average degree of concern on a 7-point scale (from 0: *not bothering me at all* to 6: *bothers me greatly*) is 5.16.

At the conclusion of every initial intake, the IP and the patient defined the integrative medicine treatment goals together. This shared decision-making was regarded by the IP as the climax of the meeting that determined which objectives were both acknowledged and accepted by the patient and could be fully regarded in the formulation of the treatment plan that followed. Common treatment goals accepted by both the patient and the IP in the 15 intakes are as follows: fatigue (12), pain (12—including neuropathy and headache), emotional distress (8—including also the caregiver's well-being), nausea/vomiting (8) and other gastro-intestinal symptoms (constipation-3, taste alteration (3), mouth sores (1), diarrhea (1), and heartburn (1)), appetite loss (5), difficulty in breathing (4), sleep (4), and others (miscellaneous).

### 3.4. Clinical Outcome Assessment

The number of integrative medicine sessions that followed the initial IP intake up to the concluding session varied from 1 (two patients participated only in IP intakes and did not attend further sessions) to 39 (mean 9.06, median 8). In total, 136 integrative medicine sessions have been recorded in the registry protocol for these patients. Typically, various CM modalities were integrated in each of the sessions and were coded according to the main CM modality and its specific technique within this modality (e.g., guided imagery is regarded as *CM code* within mind-body modality). Altogether, 383 CM codes were recorded during the 136 sessions. CM modalities that were practiced include acupuncture (in 13 patients), herbal medicine mainly traditional Arab herbs (12), manual and touch therapies (10 including acupressure, Reiki, and shiatsu), nutritional counseling with traditional Arab medicine orientation (9), mind-body-spiritual practices (7 including breathing exercises, guided imagery, and spiritual counseling and meditation), nutritional supplements (5), homeopathy (2), exercise counseling (2), and anthroposophic medicine (1).

Clinical outcome assessment was mainly based on IP's and patient's evaluation performed during the concluding session. Following this session, the IP asked the oncologist to assess the integrative treatment role in the patient's care, thus adding a third perspective to the concluding evaluation. The extent to which clinical assessment was achieved was graded on three levels: *comprehensive*—in 8 of 15 patients a 3-way perspective (IP's, patient's, and oncologist's) evaluation was obtained; *partial*—for 3 patients, evaluation was available from 2 of the 3 assessors; *deficient*—4 patients had no, or almost no, evaluation at all. These three levels of assessment comprehensiveness was often correlated with the IP's evaluation of patient's compliance (high, moderate, and low).

 Analysis of pre- and posttreatment outcomes was performed by comparing baseline and concluding session scores on the MYCAW and ESAS questionnaires. MYCAW scores which reflect patients' leading concerns, improved from 5.15 ± 0.933 to 2.05 ± 1.504 (*P* < 0.0001) (mean ± SD on a 7-point scale ranging from 0: *not bothering me at all* to 6: *bothers me greatly*). MYCAW's well being score improved from 4 ± 1.155 to 1.9 ± 1.853 (*P* = 0.015). In addition, the following symptoms improved as reflected by comparing pre- and post-ESAS scores (11-point scale ranging from 0 to 10): fatigue (6.1 ± 2.514 versus 2.9 ± 2.47, *P* = 0.024), nausea (3.9 ± 2.998 versus 1.7 ± 2.669, *P* = 0.043), depression (4.4 ± 2.951 versus 1.1 ± 1.595, *P* = 0.012), anxiety (3.6 ± 3.893 versus 1.3 ± 2.058, *P* = 0.044), appetite (4.7 ± 3.466 versus 0.9 ± 1.729, *P* = 0.012), and feeling of well-being (5.9 ± 2.601 versus 3.3 ± 2.869, *P* = 0.031). No significant statistical differences were noted regarding the ESAS subscales for pain, drowsiness, shortness of breath, and sleep quality.

Within the group of 8 patients (coded in Table 1 as patients 4–6, 8-9, 11-12, 15) with comprehensive assessment and high compliance, MYCAW scores reflected improvement in regard to nausea/vomiting (4 patients), fatigue (3), and emotional distress (3). Improvement in ESAS scores was more evident for pain (6), fatigue (6), nausea (5), anxiety (5), and depression (4). Patients' narrative evaluation as obtained in the MYCAW questionnaire administered in the concluding session emphasized the following themes: (a) improved acceptance of natural remedies (e.g., patient 4: “I feel better with natural remedies”); (b) a sense of well-being and empowerment (e.g., patient 5: “Following acupuncture I feel stronger”; patient 11: “Treatment with needles and breathing exercises gave me more strength… more energy”); (c) increased symptom control (reported by all 8 patients); (d) calming effect of the treatment (e.g., patient 6: “Acupuncture relieved pain and calmed me”; patient 12: “Acupuncture releases the body and reduces agitation”).

Within the group with deficient assessment and low compliance (4 patients coded as 1, 2, 7, and 14), two of the patients attended only one session (IP intake), whereas the other two attended 2 to 3 sessions. The information regarding the noncompliance of this group of patients is indirect and based on either a telephone interview with the independent researcher following the initial IP intake (patient 4 reporting reluctance towards needle insertion) or interviews with the patients' oncology nurse (e.g., patient 7: “Although the patient had faith in CM, she was frustrated by the dramatic deterioration in her health and felt that neither chemotherapy nor CM improved her condition”; patient 14: “The patient ignored the severity of her illness. It seems that QOL is not the most important theme for her; she just wished to “taste” CM but not make full use of it”).

Last but not least, the group of 3 patients with partial assessment and moderate to high compliance (patients coded as 3, 10, and 13) may add insight to the perceptions of the other two groups. The number of integrative sessions in this group ranged from 8 to 16 per patient. In contrast to the deficient assessment group, evaluation was available but incomplete. With two of the patients, clinical improvement was either implicit (patient 3 reporting sleep improvement following acupuncture) or explicit (patient 13 reporting that “following needling I felt better in breathing and returned a different person”). In contrast, clinical evaluation of patient 10 is contradictory, as evident in the simultaneous improvement and worsening in MYCAW and ESAS symptoms and the low score in the oncologist's evaluation.

### 3.5. Discussion

In this study, we present our experience with Arab patients referred to the IOP with the aim of improving their well-being during chemotherapy for either localized or advanced cancer. The main question we encounter daily is how optimal our communicational and clinical approach is in meeting the needs of Arab patients with cancer who are being treated in the IOP. Our intention was not to compare such aspects between Arab and Jewish patients but rather to understand and acknowledge cross-cultural barriers that potentially hamper optimal integrative care. The hypothesis that potential barriers do exist in provision of complementary therapies among the Arab population was recently supported by Keshet and Ben-Arye who surveyed 58 HMO-related complementary medicine clinics in north Israel [18].

In our study, we cautiously suggest that a disturbing gap exists between the percentage of Arab patients referred to nurse oncology intakes (19.4%) and those referred ultimately to the IOP (only 7.4% were finally enrolled in the registry protocol), as compared to non-Arab patients.

Although the NOI data includes only i.v. chemotherapy-naive patients and does not necessarily reflect patients treated with oral chemotherapy or for recurrent disease or in a palliative context, the gap between the 103 Arab patients admitted to NOI in the 2-year period and the small group of 15 patients actually enrolled in the registry protocol necessitates further contemplation. What are the reasons for this referral bias? Although further studies are needed to answer this query, four explanatory factors may be hypothesized: (1) patient-related factors (e.g., lack of patient's interest or belief that CM is beneficial during chemotherapy); (2) culturally-dependent factors (e.g., health-belief model that views cancer treatment in the context of “cure” and survival extension, rather than focusing on QOL aspects); (3) HCP-related factors (e.g., OS health providers speculating that CM may be less appropriate for Arab patients); (4) HMO-related factors (e.g., limited access of Arab patients that live in zone 3, far from Haifa, to the IOP; lack of Arab HCPs within the OS and the IOP staff, which limits communication and complicates matching expectations regarding CM). 

The question of barriers in providing integrative care to Arab patients is multifaceted (Table 2). We initiated the registry protocol and gained a preliminary unsatisfactory experience with the first 3 Arab patients (coded 1–3). As we gained more experience, we acknowledged that goodwill, openness, and sympathy to the needs of the Arab patient are not sufficient to catalyze a breakthrough. The tipping point was established when we understood that a cross-cultural dialogue needs at least two partners to embark on a journey. We understood that the IP-patient interaction mirrors a more complex cultural interaction between individual- and collective-oriented perspectives. On a practical level, we learned that patients may view CM modalities not only by an efficacy-safety scale (e.g., Does it work? Is it safe?) but also as metaphors and gestures (e.g., the invasiveness of the acupuncture needle or the calmness and feeling of contentment induced by touch). Thus, our experience has taught us that we need our patients to discover their needs alongside our own bias. Furthermore, we learned how patient-tailored treatment necessitates both skill and modesty to determine the appropriateness and sequence of treatment with herbs, touch, breathing, or needles. The establishment of trust between the patient and the IP/CM practitioner is the key element in modeling the therapeutic plan. Barriers such as the patient's reluctance to experience unfamiliar CM modalities (e.g., acupuncture, massage, and guided imagery) should not be ruled out in advance but perhaps could determine the sequence of CM modalities suggested along the course of treatment. As we gained more experience with Arab patients, we learned that herbal and nutritional counseling should typically be prioritized as the first CM modality of choice, which then can facilitate trust and openness towards additional modalities. Moreover, within each CM modality we were able to identify a scale of techniques ranging from “acceptable” to “odd” (e.g., within the mind-body modality, we typically started with a breathing exercise, moving gradually, in following sessions, to suggest closing the eyes, guided imagery, deeper meditation, etc.). Gender is another trust-dependent factor that may hamper patients' willingness to experience CM, which includes the following considerations: a mismatch of patient's and IP/CM practitioner's gender (typically female patient and male IP), the presence of another person in the room from the same or opposite sex (such as the patient's spouse, relative or another CM practitioner), and immodesty as a challenge determined either by the CM procedure (e.g., acupuncture in the knee area) or the patient's cultural/religious values, or both. As our clinical and communicational skills developed, we learned to acknowledge the complex of cultural considerations, discover our own limitations in understanding “the other” and “ourselves” in the cross-cultural equation, and become increasingly committed to the needs, concerns, and hopes of our patients who speak Arabic, Hebrew, and the many other languages spoken in our region's contemporary “Tower of Babel”.

### 3.6. Recommendations and Practice Implications

We support the following recommendations (see Table 2) aimed at improving accessibility and motivation of Arab patients to seek integrative supportive care in our oncology service. We believe that these recommendations may also be beneficial in other integrative settings in the West that provide supportive cancer care to patients from cultural minorities.

1. Location and accessibility of the integrative oncology center is a pivotal aspect, and therefore, IOP should be operated geographically within the minority population.
2. The integrative medicine staff should include a CM practitioner from the cultural minority in order to identify patients' cultural-related expectations, concerns, and barriers, with the aim of bridging the gap between traditional, complementary, and conventional agendas regarding cancer supportive care.
3. Developing integrative modalities that will resonate more with traditional medicine especially regarding the use of herbs and nutrition in relieving chemotherapy side effects and improving QOL.
4. Raising IPs' and CM practitioners' awareness of the patient's cultural and religious codes and beliefs (e.g., appropriateness of applying manual therapies (including massage and acupuncture) that may be interpreted by patients as immodest).
5. Close monitoring of the patient's compliance by improving IP-oncologist-nurse-social worker communication regarding the patient's difficulties and barriers to receiving thorough integrative care.
6. Initiating structured communication with the patient's family physician, who often operates within the cultural milieu of the patients, care givers, and the extended family circle.

### 3.7. Study Limitations and Recommendations for Following Studies

This study is limited by several considerations that may restrict the generalization of our findings to other societies and clinical settings. The group of 15 Arab patients in our registry protocol is small and lacks a control group not receiving integrative care. Thus, clinical outcomes reported in this paper may not strictly reflect the specific effects of CM intervention but also the complex interactions among the following factors: natural history of the disease and treatment-related effects (improvement as well as deterioration caused by chemotherapy) on patients' quality of life, clinical natural history (e.g., improvement of surgery-related symptoms along the course of time, disease progression causing QOL worsening, etc.), anxiety relief following the patient's adjustment to treatment, and nonspecific effects of his/her interaction with the IP and CM practitioners (e.g., attention, empathy, professionalism, etc.). However, taking this limitation into account, the comprehensiveness of our methodology provides us with a perspective of real-life patient-tailored settings and the ability to interpret social, cultural, and clinical findings in a broad and complex context, though the researcher's subjectivity should be kept in mind as potential bias. Another limitation is the lack of data regarding Arab patients with recurrent disease or during palliative care who potentially could have been referred to the IOP. Missing data is also a limiting aspect concerning the registry protocol patients with low compliance. The current study lacks sufficient qualitative research that could shed light on the motives of those Arab patients who discontinue treatment or, on the other hand, those who were highly compliant. Finally, this study is limited to the local features of the Arab community in northern Israel and the local characteristics of the CHS oncology service in Haifa, and the Integrative Oncology Program operated within the OS.

## 4. Conclusions

Barriers to integration of CM in the supportive care of Arab patients in northern Israel are multifaceted and include cross-cultural and institutional factors that influence referral and contribute to compliance and clinical outcomes. Bridging cultural gaps and traditional values with regard to CM can assuage patients' concerns and, ultimately, facilitate an enhanced integrative approach to symptom control resulting in improved quality of life.

## Figures and Tables

**Table 1:** Demographic characteristics of Arab patients enrolled in the integrative registry protocol within the oncology service (OS) in the Lin Medical Center, Haifa.

Patient's code	Age	Gender	Religion	Settlement type and zone*	Cancer site	Oncology status (and chemotherapy setting)
1	59	F	Christian	Urban 1	Breast	Localized (adjuvant)
2	44	F	Muslim	Urban 3	Breast	Localized (adjuvant)
3	62	F	Christian	Urban 3	Breast	Advanced (palliative)
4	47	M	Druze	Rural 3	Mesothelioma	Advanced (palliative)
5	46	F	Druze	Rural 3	Stomach	Localized (adjuvant)
6	45	F	Druze	Rural 3	Breast	Localized (adjuvant)
7	44	F	Druze	Semiurban 3	Unknown	Advanced (palliative)
8	62	F	Christian	Urban 3	Breast	Localized (adjuvant)
9	38	F	Muslim	Urban 3	Breast	Localized (adjuvant)
10	22	M	Muslim	Rural 3	Testicular	Localized (adjuvant)
11	65	F	Muslim	Urban 3	Bladder	Localized (adjuvant)
12	67	M	Muslim	Urban 1	Colon	Localized (adjuvant)
13	67	F	Muslim	Semiurban 2	Lung	Advanced (palliative)
14	42	F	Christian	Urban 1	Colon	Advanced (palliative)
15	77	M	Christian	Urban 1	Colon	Advanced (palliative)

*Distance from the oncology service in Haifa is classified according to zones as follows: zone 1: city of Haifa; zone 2: up to 20 kilometers from Haifa; zone 3: more than 20 kilometers from Haifa.

**Table 2:** Potential barriers to CM integration in supportive cancer care of Arab patients in northern Israel and recommendations for bridging the barriers.

Potential Barrier	Recommendation	Practical implications
Geographical factor: 68% of the Arab patients receiving chemotherapy reside >20 km from the IOP in Haifa OS	Opening a second site of IOP activity in Haifa periphery (zone 2 or 3)	Minimizing distance-bias may help patients to overcome initial hesitations regarding the first IP visit and enable them to attend weekly CM sessions
Not having an Arab CM practitioner in the IOP staff	Inclusion of an Arab CM practitioner (preferably a dual practitioner) in the IOP	Improving verbal communication with patients, enhancing the IOP attentiveness to their needs, and concerns and promoting development of traditional Arab-oriented therapies
Gap between patients' expectations and IOP objectives and CM repertoire	Increasing IP awareness of patients' expectations; developing integrative modalities that will resonate more with traditional Arab medicine	Matching patients' expectations with IP goals of treatment is essential and should be continuously monitored, especially with regard to QOL-oriented care rather than “attacking” cancer cells
Suboptimal matching of CM modalities to patients' cultural and religious codes and beliefs	Raising the IP's and CM practitioner's awareness of cultural and religious codes within the Arab society	The IOP staff need to consider cultural appropriateness of certain CM modalities (e.g., touch), reluctance concerning unfamiliar treatments (e.g., acupuncture, guided imagery), and gender issues (patient treated by a CM practitioner of the opposite sex, presence of another person in the room, etc.)
Suboptimal communication between the IOP and the other OS sectors	Improving IP-oncologist-nurse- social worker communication in order to enhance coordinated comprehensive care	Closer monitoring of patient compliance may also reveal the patient's difficulties and barriers to seeking integrative care
Lack of communication with the patient's family physician (FP)	Initiating a structured form of communication with the patient's FP via summary letter e-mailed from the patient's medical file	The FP often operates within the cultural milieu of the patients, care givers, and the extended family circle Integrative care-oriented medical education courses are needed to familiarize FPs with the IOP activity
